# Bona fide colour: DNA prediction of human eye and hair colour from ancient and contemporary skeletal remains

**DOI:** 10.1186/2041-2223-4-3

**Published:** 2013-01-14

**Authors:** Jolanta Draus-Barini, Susan Walsh, Ewelina Pośpiech, Tomasz Kupiec, Henryk Głąb, Wojciech Branicki, Manfred Kayser

**Affiliations:** 1 Institute of Forensic Research, Section of Forensic Genetics, Kraków, Poland; 2 Department of Forensic Molecular Biology, Erasmus MC University Medical Center Rotterdam, Rotterdam, The Netherlands; 3 Department of Anthropology, Institute of Zoology, Faculty of Biology and Earth Sciences, Jagiellonian University, Kraków, Poland; 4 Department of Genetics and Evolution, Institute of Zoology, Faculty of Biology and Earth Sciences, Jagiellonian University, Kraków, Poland

**Keywords:** Skeletal remains, Forensic DNA phenotyping, Eye colour, Hair colour, HIrisPlex, Ancient DNA, Human appearance, Władysław Sikorski

## Abstract

**Background:**

DNA analysis of ancient skeletal remains is invaluable in evolutionary biology for exploring the history of species, including humans. Contemporary human bones and teeth, however, are relevant in forensic DNA analyses that deal with the identification of perpetrators, missing persons, disaster victims or family relationships. They may also provide useful information towards unravelling controversies that surround famous historical individuals. Retrieving information about a deceased person’s externally visible characteristics can be informative in both types of DNA analyses. Recently, we demonstrated that human eye and hair colour can be reliably predicted from DNA using the HIrisPlex system. Here we test the feasibility of the novel HIrisPlex system at establishing eye and hair colour of deceased individuals from skeletal remains of various *post-mortem* time ranges and storage conditions.

**Methods:**

Twenty-one teeth between 1 and approximately 800 years of age and 5 contemporary bones were subjected to DNA extraction using standard organic protocol followed by analysis using the HIrisPlex system.

**Results:**

Twenty-three out of 26 bone DNA extracts yielded the full 24 SNP HIrisPlex profile, therefore successfully allowing model-based eye and hair colour prediction. HIrisPlex analysis of a tooth from the Polish general Władysław Sikorski (1881 to 1943) revealed blue eye colour and blond hair colour, which was positively verified from reliable documentation. The partial profiles collected in the remaining three cases (two contemporary samples and a 14th century sample) were sufficient for eye colour prediction.

**Conclusions:**

Overall, we demonstrate that the HIrisPlex system is suitable, sufficiently sensitive and robust to successfully predict eye and hair colour from ancient and contemporary skeletal remains. Our findings, therefore, highlight the HIrisPlex system as a promising tool in future routine forensic casework involving skeletal remains, including ancient DNA studies, for the prediction of eye and hair colour of deceased individuals.

## Background

Skeletal remains represent a unique type of biological material. Due to their unique features, bones and teeth can be resistant to degradation and, depending on the environmental storage conditions, can provide a good source of DNA suitable for genetic analysis thousands of years after an organism’s death. DNA analysis of ancient bones and teeth is used to explore various aspects of a species’ history, including archaic and modern humans. For instance, the application of next generation DNA sequencing (NGS) technologies to ancient human bones has allowed the establishment of whole genome information on Neanderthals [1] and Denisovans [2]. These studies not only revealed that Denisovans represent a distinct archaic human taxon [2], which was impossible to conclude from the finger bone found as its only evidence, but also highlighted that a genetic admixture likely occurred between both archaic human taxons and modern humans, respectively [1-4].

Contemporary bones and teeth are frequently used in dedicated forensic laboratories dealing with missing person identification cases, including disaster victim identification. Although DNA extraction from skeletal remains is very complex and time consuming with limited possibilities for automation, in forensic science teeth and bones constitute the most suitable sources of DNA for individual identification from heavily decomposed bodies. Forensic DNA analysis of contemporary bones and teeth typically concerns autosomal short tandem repeats (STRs, or microsatellites) for direct match identification with antemortem samples of the same individual, or via STR profile similarities with close putative relatives [5,6]. Mitochondrial (mt) DNA and Y-chromosomal DNA analysis also represent two genetic marker systems that allow profile matching not only with close but also with distant putative maternal and paternal relatives, respectively [7]. Furthermore, there seems to be continual interest in the DNA analysis of remains from famous historical individuals as seen with studies about the last Russian Tsar Nikolaus II (1868 to 1918) and his close family [8-11], the Polish astronomer Nicolaus Copernicus (1473 to 1543) [12], the Italian poet and founder of humanism Francesco Petrarca (1304 to 1374) [13], or perhaps even the evangelist Luke [14] to name but a few examples.

One interesting aspect of ancient and modern skeletal analysis, which has been largely overlooked thus far, is the reconstruction of the deceased person’s appearance. In ancient DNA analysis the prediction of particular externally visible characteristics (EVCs) not reflected in skeletal features, such as eye and hair colour, is the only way to get insights into how extinct species may have looked. In contemporary skeletal analysis, such as forensic cases, EVC prediction from DNA can be useful in providing leads in police investigation to reveal the identity of a deceased person if other and more direct means (such as STR, mtDNA and Y-DNA profiling) are non-informative. In recent years, researchers have started seeing increased success in DNA-based EVC prediction, a field sometimes referred to as Forensic DNA Phenotyping (FDP) or DNA intelligence [15,16]. From all human externally visible characteristics (besides sex), pigmentation traits, particularly eye and hair colour, are currently the most promising EVCs for DNA prediction. This is due to limited genetic complexity coupled with limited environmental impact that has led to accumulative success in exploring the genetic basis of eye and hair colour via candidate gene and genome-wide association studies [17-22]. From these association studies, highly predictive eye and hair colour DNA markers have been identified [23-33].

Previous ancient DNA studies have suggested that Neanderthals may have had light skin and reddish hair colour due to a particular non-synonymous variation in *MC1R*[34]. Genetic variation in *MC1R* causes light skin colour and red hair colour in modern humans [35], albeit via different DNA variants than found in Neanderthals in that study. Recently, whole genome sequencing (WGS) data from bones of Neanderthals, Denisovans and modern humans from different time periods have been established using NGS technologies and in part have been explored for the prediction of pigmentation (and other visible and non-visible) traits [36-38]. However, the establishment of WGS data from bones, though shown to be feasible via NGS, still comes with a stiff price tag not easily affordable by most laboratories. Furthermore, WGS represents a type of data overkill when one is only interested in specific traits determined by a limited number of genes, such as eye and hair colour. Lastly, WGS comes with the disadvantage that if the overall genome coverage is low, (which often is the case in aDNA NGS/WGS studies), the quality for the particular single nucleotide polymorphisms (SNP) genotypes required for prediction can be low with consequences for the final trait prediction. What, therefore, would be welcomed are more dedicated and targeted approaches allowing the retrieval of high quality EVC information, such as eye and hair colour, from ancient and contemporary human skeletal remains in an effective manner, such as multiplex analyses.

Previously, we developed and forensically validated the IrisPlex system for reliable and accurate DNA prediction of blue and brown eye colour, which includes a sensitive single multiplex assay of six highly eye colour-predictive SNPs and an eye colour prediction model based on genotype and phenotype data of many thousands of individuals from various regions in Europe [24-26]. An independent test dataset of > 3,800 individuals from seven European sampling sites has shown that the IrisPlex system provides on average 94% individual-based accuracy for blue and brown eye colour prediction, respectively [26]. In an earlier study, some pigmentation associated SNPs had been combined in a multiplex assay and tested successfully on ancient bone samples [39]. However, the predictive value of some of the SNPs considered in that study is low and lower than the SNPs that only became available via subsequent studies. In particular, they typed only two of the six best eye colour predictors included in the IrisPlex assay. Recently, a highly sensitive single multiplex genotyping assay has been developed, together with model-based tools supported by large genotype and phenotype databases, allowing parallel prediction of eye and hair colour from a DNA sample [40]. This so called HIrisPlex system targets 24 eye and/or hair colour predictive DNA polymorphisms (including all 6 IrisPlex SNPs) and provides individual prediction probabilities for eye and hair colour categories as well as hair colour shade categories [40]. As shown recently, this approach allows the prediction of hair colour with average individual-based prediction accuracies of 69.5% for blond, 78.5% for brown, 80% for red and 87.5% for black hair colour on an independent test set of > 300 individuals from three European sampling sites [40]. Furthermore, it was previously shown by analysing a worldwide sample set (HGDP-CEPH) that IrisPlex/HIrisPlex eye and hair colour prediction works reliably independent of knowledge on bio-geographic ancestry [24,40]. In the present study we test the feasibility of predicting eye and hair colour of deceased individuals from DNA analysis of ancient and contemporary teeth and bones using the novel HIrisPlex system.

## Methods

### Bone and teeth samples

The specimens subjected to DNA analysis were comprised of 21 teeth and 5 bones. Samples S1 to S3 were collected from contemporary human remains approximately one to two years after death as estimated from medico-legal and criminalistic examination. S4 was a tooth collected from modern remains of an incomplete skeleton that rested in soil; it was impossible to estimate an approximate age of the remains but anthropological examination showed they were seriously decayed. Serious degradation was also present in cases S5 to S10, which were contemporary human remains found in a cave. S5 was a fragment of a femur, S6 was a fragment of a tibia, S7 was a fragment of a humerus, S8 was a fragment of another humerus, S9 was a tooth collected from a mandible and S10 was a part of a skull; they were all found in one location in a cave situated in Dolina Będkowska Landscape Park near Kraków. S11 was a tooth collected from the exhumed remains of General Władysław Sikorski; the exact date of his death is known (1943) and thus, we know that the analyzed tooth was 69 years of age. S12 to S23 were samples collected from skulls which in 1942 were subjected to bleaching and afterwards kept in a museum in Austria until 1998. S24 was a tooth of a mysterious woman from a Benedictine crypt in Tyniec near Kraków, which according to historical evidence comes from the 12^th^ to 14^th^ century. S25 and S26 were teeth picked up from skeletons found in the St. Andrew Church in Kraków and dated based on historical markers to come from the 14^th^ century.

### Precautions taken to avoid contamination

Multifarious efforts were undertaken in the laboratory in order to prevent contamination. Full protective clothing and separate working localities for extraction, amplification and SBE reaction setup were used. All DNA extractions were done in a separate room reserved for low template DNA samples. DNA extraction and PCR were performed in clean room facilities with Laminar Air Flow benches equipped with HEPA filters (Thermo Fisher Scientific, Hudson, New Hampshire, USA). All working areas and equipment were regularly UV-irradiated and cleaned with bleach. In total, 10 negative extraction controls were included. Each presented case was subjected to separate DNA extraction, except for samples S12 to S23 where three negative extraction controls were used. Negative controls were also included for each PCR amplification performed. All samples were first subjected to STR analysis which showed no traces of DNA admixture that could indicate contamination. Moreover, in order to exclude any possibility of internal contamination, STR (AmpFℓSTR® NGM™ kit (Applied Biosystems, Foster City, CA, USA)) and HIrisPlex profiles were determined for all 10 members of the laboratory staff. No match was found for any of the samples analysed in both STR and HIrisPlex profiles.

### DNA extraction

DNA was isolated using standard organic extraction protocols. Before extraction, bone material comprising teeth or fragments of femurs was subjected to a purification and decontamination procedure. Samples were treated with 15% bleach, repeatedly shaken with 70% ethanol and distilled water, and while drying were subjected to UV irradiation. The entire tooth or bone fragments were then pulverized using FreezerMill 6750 (SPEX CertiPrep Metuchen, New Jersey, USA) and subjected to the extraction procedure. When available, 3 g (in case of tooth samples 1 to 1.5 g) of bone powder were incubated overnight in a water bath set at 56°C with 3 mL of buffer composed of 0.5 M EDTA (Sigma-Aldrich Corporation, St. Louis, Missouri, USA) and 10% SDS (Amresco LLC, Solon, Ohio, USA) with the addition of 225 μL proteinase K (10 mg/mL) (Sigma-Aldrich Corporation, St. Louis, Missouri, USA) and 120 μL 1 M DTT (Amresco LLC, Solon, Ohio, USA). The samples were then subjected to double extraction with a phenol-chloroform-isoamyl alcohol (Sigma-Aldrich Corporation, St. Louis, Missouri, USA) and concentrated after extraction using Amicon Ultra 4 – 30k columns and Microcon 100 (Millipore, Carrigtwohill, Cork, Ireland) to the final volume of 60 to 70 μl in all cases. Negative extraction controls were used to check the purity of the used chemicals and consumables.

### DNA quantification

DNA concentration was assessed on a 7500 Real Time PCR system using *Quantifiler*™ Human DNA Quantification Kit (Applied Biosystems, Foster City, CA, USA), following the protocol recommended by the manufacturer.

### Genotyping with the HIrisPlex assay

Multiplex genotyping was performed as described in detail recently [40]. The HIrisPlex assay relies on simultaneous PCR amplification followed by simultaneous SNaPshot primer extension (Applied Biosystems, Foster City, CA, USA) (or minisequencing) of 24 DNA polymorphisms previously established with predictive value on human eye and hair colour, namely N29insA, rs11547464, rs885479, rs1805008, rs1805005, rs1805006, rs1805007, rs1805009, Y152OCH, rs2228479, rs1110400 – all from the *MC1R* gene, rs12913832 from *HERC2*, rs12203592 from *IRF4*, rs1042602 and rs1393350 from *TYR*, rs4959270 from *EXOC2*, rs28777 and rs16891982 from *SLC45A2*, rs683 from *TYRP1*, rs1800407 from *OCA2*, rs2402130 and 12896399 from *SLC24A4*, rs12821256 from *KITLG* and rs2378249 from *ASIP*. All samples were genotyped at least twice.

### Prediction of eye and hair colour with the HIrisPlex system

Eye colour inference from the genotyping results of the six SNPs (the same as used in IrisPlex) was obtained via model-based prediction using a database of thousands of Europeans and a convenient Microsoft Excel macro (Microsoft Corporation, Redmont, Washington, USA) published previously [24,26]. Hair colour inference from the genotypes of 22 SNPs was obtained via model-based prediction using a database of > 1,500 Europeans with an Excel macro and following a prediction guide that not only considers hair colour categories but also hair colour shade for the final assessment as introduced recently [40].

## Results and discussion

Using a standard organic extraction protocol, we purified genomic DNA from 21 teeth aged between 1 year and approximately 800 years (12^th^ century) and 5 contemporary bones. All samples were subjected to multiple genotyping using the HIrisPlex assay and to model-based eye and hair colour prediction from the combined HIrisPlex genotypes using large genotype/phenotype databases described elsewhere [24,26,40]. It is also worth emphasising that in studies of skeletal remains, unlike some other human characteristics, pigmentation cannot be concluded from basic anthropological research and thus reliable DNA prediction of eye and hair colour shall be particularly useful. The ascertained HIrisPlex genotypes for all 24 DNA polymorphisms and all 26 samples analysed are presented in Table 1. The derived eye and hair colour probabilities, the predicted eye and hair colours and the accuracy probability values of predicted eye and hair colours for all 26 individuals are provided in Table 2. Although in all but one of the samples analysed the phenotypic eye and hair colour of the deceased individuals analysed were unknown, the previously obtained accuracy estimates from large numbers of individuals with known phenotype and genotype information provide reasonable confidence that the eye and hair colour phenotypes predicted in this study match the true phenotypes of the individuals before death. However, because previous studies showed that the estimated probabilities and attached accuracies are higher for eye than for hair colour categories, more confidence can be expected in eye colour over the hair colour prediction results obtained here.

**Table 1:** HIrisPlex genotyping results together with sample age and starting DNA amount for the 26 samples tested

**Sample ID**	**Sample/postmortem age**	**Starting template DNA (ng)**	**Genotype**																							
			1	2	3	4	5	6	7	8	9	10	11	12	13	14	15	16	17	18	19	20	21	22	23	24
**S1**	T/CR	0.6	C	G	C	C	G/T	C	C	G	C	G	T	A	G	A	C/A	C/T	G	G	A	C/T	T	T	C	G/T
**S2**	T/CR	1.7	C	G	C/T	C	G	C	C	G	C	G	T	A	G	A	C/A	C	G	G	A	C/T	T	T	C/T	G/T
**S3**	T/CR	1.2	C	G	C/T	C	G	C	C	G	C	G	T	A	G	G/A	C/A	C/T	G/T	G	G	C/T	T	G	C/T	G/T
**S4**	T/C	0.3	-	G	C	C/T	G	C	C	G	C	G	T	A	G	A	C/A	C	G/T	G	A	C	T	G/T	C	T
**S5**	B/C	U^a^	C	G	C	C	G	C	C	G	C	G	T	C	C	A	C/A	C/T	G/T	G	A	C	T	T	C	G
**S6**	B/C	0.3	C	G	C	C	G	C	C	G	C	G	T	A	G	A	C	C	G/T	G	A	T	T	G/T	C	T
**S7**	B/C	0.15	-	G	C	C	G	C	C	G	C	G	T	C	C	A	C/A	C/T	G/T	G	A	C	T	T	C	G
**S8**	B/C	U	C	G	C	C	G	C	C	G	C	G	T	C/A	G/C	A	C/A	C	G	G	A	C/T	T	G	C	G/T
**S9**	B/C	0.5	C	G	C	C	G	C	C	G	C	G	T	C/A	G/C	A	C/A	C/T	G	G	A	C	T	T	C/T	G/T
**S10**	B/C	0.1	C	G	C	C	G	C	C	G	C	G	T	C/A	G/C	A	C	C	G	G	G/A	T	T	G/T	C/T	G/T
**S11**	T/2WW	0.6	C	G	C	C/T	G	C	C	G	C	G	T	A	G	A	A	C	G/T	G	A	C	T	T	C	T
**S12**	T/2WW	2	C	G	C	C/T	G	C	C	G	C	G	C/T	A	G	A	C	C	G	G	A	C	T	G/T	C/T	G/T
**S13**	T/2WW	0.3	C	G	C	C/T	G	C	C	G	C	G	T	A	G	A	A	C	G	G	A	C/T	T	T	C	G
**S14**	T/2WW	0.2	C	G	C	C	G	C	C	G	C	G	T	A	G	A	C/A	C	T	G	G/A	C	T	G	C/T	T
**S15**	T/2WW	1.2	C	G	C	C	G	C	C	G	C	G	T	A	G	A	A	C	G/T	G	G/A	C	T	G	C	G
**S16**	T/2WW	0.1	C	G	C	C	G	C	C	G	C	G	T	A	G	A	C/A	C	G	G	A	C	T	T	C	T
**S17**	T/2WW	0.8	C	G	C	C	G	C	C	G	C	G	T	A	G	A	C/A	C	G	G	A	C/T	T	G/T	C/T	G/T
**S18**	T/2WW	2.8	C	G	C	C/T	G	C	C	G	C	G	T	A	G	A	C/A	C	G/T	G	A	C	C/T	G/T	C/T	G/T
**S19**	T/2WW	0.8	C	G	C	C	G	C	C	G	C	G	T	A	G	A	A	C	G	G	A	C	T	T	C	T
**S20**	T/2WW	0.4	C	G	C	C	G	C	C	G	C	G	T	A	G	G/A	C	C	G/T	G	A	C/T	T	G/T	C/T	G/T
**S21**	T/2WW	0.3	C	G	C	C	G	C	C	G	C	G	T	A	G	A	C/A	C	G	G	A	C	T	G/T	C/T	T
**S22**	T/2WW	0.7	C	G	C	C	G	C	C	G	C	G	T	A	G	G	A	C	G	G	G	C	C/T	G	C	T
**S23**	T/2WW	1.3	C	G	C	C	G	C	C	G	C	G	T	A	G	A	C/A	C	G/T	G	A	C/T	T	G/T	C	T
**S24**	T/XII	0.03^b^	C	G	C	C	G	C	C	G	C	G/A	T	A	G	A	C/A	C	G	G	A	C/T	T	G/T	T	G/T
**S25**	T/XIV	0.01^b^	-	G	C	C	-	C	T	G	C	-	T	A	G	A	C/A	C	T	G	G	C	T	G	C/T	G/T
**S26**	T/XIV	0.16	C	G	C	C	G	C	C	G	C	G/A	T	A	G	A	C/A	C	T	G	A	C	T	G/T	C	G/T

^a^ U - DNA concentration was not determined due to complete inhibiton of *Quantifiler*™ Human DNA Quantification Kit reaction.

^b^ DNA concentration may be higher as significant inhibition of *Quantifiler*™ Human DNA Quantification Kit reaction was observed. DNA markers are shown in the following order from 1 to 24: 1-N29insA, 2-rs11547464, 3-rs885479, 4-rs1805008, 5-rs1805005, 6-rs1805006, 7-rs1805007, 8-rs1805009, 9-Y152OCH, 10-rs2228479, 11-rs1110400, 12-rs28777, 13- rs16891982, 14- rs12821256, 15-rs4959270, 16- rs12203592, 17- rs1042602, 18-rs1800407, 19-rs2402130, 20-rs12913832, 21- rs2378249, 22-rs12896399, 23-rs1393350, 24- rs683. Samples S5 and S7 come from the same individual. In the column Sample/postmortem age: T indicates tooth and B indicates bone. CR-indicates contemporary and relatively fresh specimen (one to two years postmortem age); C-indicates contemporary but older than CR; 2WW-indicates samples from the World War 2; XII and XIV-indicate centuries assumed from historical evidence.

**Table 2:** HIrisPlex-based eye and hair colour prediction results for the 26 samples tested

**Sample**	**Probability values of hair colour categories**	**Probability values of hair colour shade**	**Inferred most likely hair colour**	**Accuracy probability value of predicted hair colour based on a >300 European test set**	**Probability values of eye colour categories**	**Inferred most likely eye colour**	**Accuracy probability value of predicted eye colour based on a >3800 European test set**
**S1**	Brown 0.367	Light 0.268	Dark Brown	78.5%	Blue 0.317	Brown	87.5%
	Red 0.002	Dark 0.732			Int. 0.193		
	Black 0.499				Brown 0.490		
	Blond 0.133						
**S2**	Brown 0.246	Light 0.655	Dark Blond/Brown	78.5%	Blue 0.306	Brown	91%
	Red 0.001	Dark 0.345			Int. 0.142		
					Brown 0.552		
	Black 0.326						
	Blond 0.427						
**S3**	Brown 0.496	Light 0.215	Dark Brown	78.5%	Blue 0.190	Brown	90.4%
	Red 0.001	Dark 0.785			Int. 0.271		
	Black 0.406				Brown 0.539		
	Blond 0.098						
**S4**	Brown 0.064	Light 0.976	Light Blond	69.5%	Blue 0.919	Blue	97.4%
	Red 0.048	Dark 0.024			Int. 0.048		
	Black 0.025				Brown 0.033		
	Blond 0.864						
**S5**	Brown 0.251	Light 0.020	Black/Dark Brown	87.5%	Blue 0.706	Blue	94%
	Red 0.000	Dark 0.980			Int. 0.117		
	Black 0.729				Brown 0.177		
	Blond 0.020						
**S6**	Brown 0.227	Light 0.171	Dark Brown	78.5%	Blue 0.002	Brown	99%
	Red 0.000	Dark 0.829			Int. 0.026		
	Black 0.636				Brown 0.972		
	Blond 0.136						
**S7**	Brown 0.282	Light 0.030	Black/Dark Brown	87.5%	Blue 0.706	Blue	94%
	Red 0.000	Dark 0.970			Int. 0.117		
	Black 0.690				Brown 0.177		
	Blond 0.028						
**S8**	Brown 0.246	Light 0.196	Dark Brown	78.5%	Blue 0.024	Brown	95.6%
	Red 0.000	Dark 0.804			Int. 0.083		
	Black 0.594				Brown 0.892		
	Blond 0.160						
**S9**	Brown 0.324	Light 0.212	Dark Brown	78.5%	Blue 0.911	Blue	97.4%
	Red 0.001	Dark 0.788			Int. 0.057		
	Black 0.538				Brown 0.032		
	Blond 0.136						
**S10**	Brown 0.153	Light 0.016	Black	87.5%	Blue 0.001	Brown	99%
	Red 0.000	Dark 0.984			Int. 0.016		
	Black 0.829				Brown 0.983		
	Blond 0.017						
**S11**	Brown 0.076	Light 0.964	Light Blond	69.5%	Blue 0.950	Blue	99%
	Red 0.037	Dark 0.036			Int. 0.030		
	Black 0.035				Brown 0.020		
	Blond 0.852						
**S12**	Brown 0.044	Light 0.969	Light Blond	69.5%	Blue 0.937	Blue	97.4%
	Red 0.090	Dark 0.031			Int. 0.041		
	Black 0.049				Brown 0.022		
	Blond 0.817						
**S13**	Brown 0.180	Light 0.883	Dark Blond/Light Brown	69.5%	Blue 0.229	Brown	91.9%
	Red 0.025	Dark 0.117			Int. 0.128		
	Black 0.119				Brown 0.643		
	Blond 0.676						
**S14**	Brown 0.157	Light 0.907	Light Blond	69.5%	Blue 0.899	Blue	95.6%
	Red 0.005	Dark 0.093			Int. 0.066		
	Black 0.072				Brown 0.035		
	Blond 0.766						
**S15**	Brown 0.168	Light 0.927	Light Blond	69.5%	Blue 0.870	Blue	95.6%
	Red 0.004	Dark 0.073			Int. 0.076		
	Black 0.064				Brown 0.053		
	Blond 0.765						
**S16**	Brown 0.089	Light 0.951	Light Blond	69.5%	Blue 0.950	Blue	99%
	Red 0.002	Dark 0.049			Int. 0.030		
	Black 0.054				Brown 0.020		
	Blond 0.856						
**S17**	Brown 0.221	Light 0.696	Dark Blond/Brown	78.5%	Blue 0.207	Brown	91.9%
	Red 0.001	Dark 0.304			Int. 0.161		
	Black 0.263				Brown 0.632		
	Blond 0.514						
**S18**	Brown 0.083	Light 0.969	Light Blond	69.5%	Blue 0.937	Blue	97.4%
	Red 0.090	Dark 0.031			Int. 0.041		
	Black 0.024				Brown 0.022		
	Blond 0.802						
**S19**	Brown 0.075	Light 0.968	Light Blond	69.5%	Blue 0.950	Blue	99%
	Red 0.002	Dark 0.032			Int. 0.030		
	Black 0.039				Brown 0.020		
	Blond 0.883						
**S20**	Brown 0.246	Light 0.727	Dark Blond/Brown	78.5%	Blue 0.207	Brown	91.9%
	Red 0.001	Dark 0.273			Int. 0.161		
	Black 0.183				Brown 0.632		
	Blond 0.570						
**S21**	Brown 0.089	Light 0.951	Light Blond	69.5%	Blue 0.937	Blue	97.4%
	Red 0.002	Dark 0.049			Int. 0.041		
	Black 0.054				Brown 0.022		
	Blond 0.856						
**S22**	Brown 0.155	Light 0.934	Light Blond	69.5%	Blue 0.870	Blue	95.6%
	Red 0.008	Dark 0.066			Int. 0.076		
	Black 0.053				Brown 0.053		
	Blond 0.783						
**S23**	Brown 0.218	Light 0.696	Dark Blond/Brown	78.5%	Blue 0.150	Brown	94%
	Red 0.002	Dark 0.304			Int. 0.140		
	Black 0.248				Brown 0.711		
	Blond 0.532						
**S24**	Brown 0.202	Light 0.695	Dark Blond/Brown	78.5%	Blue 0.277	Brown	90.4%
	Red 0.002	Dark 0.305			Int. 0.179		
	Black 0.301				Brown 0.543		
	Blond 0.495						
**S25**	-	-	-		Blue 0.899	Blue	95.6%
					Int. 0.066		
					Brown 0.035		
**S26**	Brown 0.131	Light 0.918	Light Blond	69.5%	Blue 0.919	Blue	97.4%
	Red 0.007	Dark 0.082			Int. 0.048		
	Black 0.078				Brown 0.033		
	Blond 0.784						

Probability estimation and final prediction of eye colour was done according to Walsh *et al.* 2011 [24] and eye colour accuracy estimation according to Walsh *et al.* 2012 [26].

Probability and accuracy estimation and final prediction for hair colour and hair colour shade was done according to Walsh *et al.* 2012 [40].

### Contemporary samples

A number of contemporary forensic DNA identification cases remain unsolved because of no match between the evidence STR profile and a reference DNA profile from a suspect or from the forensic DNA database search. Similarly, a number of missing person cases, including disaster victim identification (DVI) cases, remain unsolved because no STR profile match with the antemortem samples, or because no informative STR profile similarities with known putative relatives can be obtained. In all such cases, EVC information inferred from the scene sample, including skeletal remains, may be valuable for the continuing investigation. Representing typical forensic casework samples, we included three teeth collected from partially or completely skeletonized bodies of approximately one to two years post mortem age. S1 was a cadaver and S2 was a skeleton, both found in small villages in different regions of Poland in the open soil. S3 was a cadaver found inside a building located in Kędzierzyn-Koźle, a small city in southern Poland. The teeth were previously subjected to standard forensic identification using STR and mitochondrial DNA profiling. Complete STR profiles (NGM™ PCR Amplification Kit or Identifiler® PCR Amplification Kit (Applied Biosystems, Foster City, CA, USA)) were obtained in each case indicating reasonably good preservation of nuclear DNA. The amelogenin gender test revealed that skeletons S1 and S3 were males and S2 a female, corroborating results of the anthropological investigation of the skeletons (data not shown). Complete HIrisPlex profiles were determined for all three samples (Table 1). For S1 to S3 the highest probability (*P*) was obtained for brown eye colour (S1 = 0.490, S2 = 0.552 and S3 = 0.539, with accuracies S1 = 87.5%, S2 = 91%, and S3 = 90.4%, Table 2). In cases S1 and S3 hair colour was predicted as dark brown (accuracy 78.5%) and in case S2 hair colour was predicted as dark blond/brown (accuracy 78.5%, Table 2). Samples S1 and S3 were not associated with any reference material and all the genetic data, including eye and hair colour inference, may be used in further investigating these cases. Sample S3 was associated with a reference sample from a putative close relative but in the course of genetic analysis the assumed hypothesis of a relationship was denied.

Occasionally, human skeletal remains are discovered coincidentally by workers or sightseers. They may be of old or young post mortem age. Sample S4 came from the human remains found in the field near Sieradz in central Poland. Anthropological examination revealed that the bones belong to a woman of about 40 years of age at the time of death. The skeletal remains may have rested in the soil for a very long time as they appeared to be seriously decayed. Two teeth were taken from the maxilla for DNA extraction. A partial NGM STR profile was determined (seven markers were genotyped successfully) and the positive amelogenin result confirmed the female origin suggested from the anthropological investigation. In this case the level of DNA concentration was critical as *Quantifiler*™ Human DNA Quantification Kit (Applied Biosystems, Foster City, CA, USA) indicated merely 7 pg/μl of human DNA. As template DNA concentration in the HIrisPlex reaction equalling approximately 30 pg was below the genotyping accuracy threshold of approximately 60 pg as previously established [40], genotyping was repeated three times. Results for all but one (86insC *MC1R*) HIrisPlex SNPs were obtained successfully. Blue eye colour was inferred with high probability (*P* = 0.919) and accuracy (*P* = 97.4), (Table 2). Blond hair color was inferred with a probability of 0.864 and light hair colour shade with a probability of 0.976 so that light blond is assumed as the most likely hair colour. However, red hair cannot be excluded because of the missing ins86A *MC1R* genotype. Although this DNA variant (indel) is very rare in the general European population (allele observed 4 times among more than 1,000 samples, data not shown), its impact on red hair colour is very strong.

Samples S5 to S10 came from human remains discovered in one of several caves located in the landscape park Dolina Będkowska near Kraków (Poland). Anthropological examination indicated that the revealed 13 skeletal elements, including pelvis, femurs, humerus, ribs, vertebras, mandible comprising two teeth and an incomplete skull, may belong to 6 individuals. The same mtDNA and STR profiles were ascertained in the femur (S5) and the humerus (S7) indicating that these bones likely belong to a single female skeleton (data not shown). Overall genetic analysis showed that in fact all remains came from only five individuals, two females (S5/S7 and S9) and three males (S6, S8 and S10). However, only a partial STR profile was determined in S7 showing more serious degradation compared with S5. Both types of bone (femur and humerus) are usually considered a good source of DNA and thus the observed difference may be coincidental. Complete 24 SNP HIrisPlex profiles were generated for all samples except for S7 where again the ins86A *MC1R* genotype was missing. The obtained data show that from all 24 HIrisPlex polymorphisms indel position 86insA in *MC1R* seems most affected by suboptimal DNA concentration and/or DNA degradation. The S7 profile was also inconsistent among three repeated analyses carried out in terms of rs4959270. From the HIrisPlex profile ascertained in sample S5 we could further conclude that drop-out (rs4959270C/A -> A) occurred in this position in sample S7 (observed twice among three repetitions) keeping with the hypothesis that indeed both samples derive from the same individual. Inferred eye and hair colours for S5 and S7 are blue and black/dark brown, respectively, with highly similar probabilities and accuracies agreeing with the idea that they were derived from the very same individual. Notably, strong PCR inhibition prevented determination of DNA concentration in samples S5 and S8 using *Quantifiler*™ Human DNA Quantification Kit (Table 1). Nevertheless, genotyping was possible in both samples showing that the HIrisPlex assay is indeed insensible to inhibition similarly to commercial STR kits used for human identification purposes. Inferred eye colour was blue for S9 (probability 0.911, accuracy 97.4%) and brown for S6, S8 and S10 (probabilities 0.972, 0.892, and 0.983, respectively; accuracies 99%, 95.6% and 99%, respectively). Inferred hair colour was dark brown for S6, S8 and S9 (accuracy 78.5%) and black for S10 (accuracy of 87.5%).

### Samples from World War II

**Figure 1:**
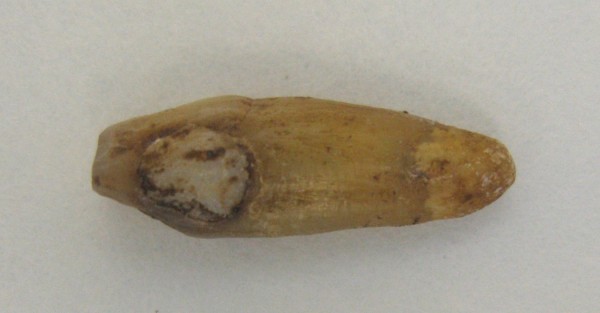
The photograph of the analysed tooth of General Władysław Sikorski (World War II).

**Figure 2:**
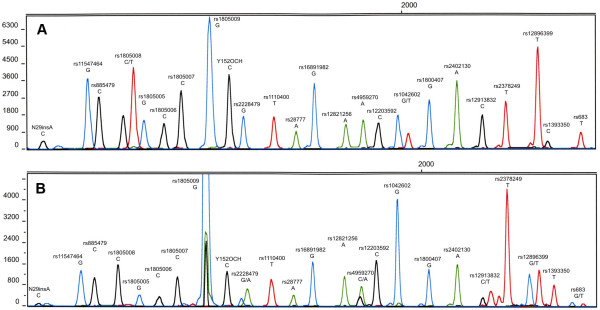
**HIrisPlex SNP electropherograms obtained from two selected teeth samples. A**) A tooth of the Polish General Władysław Sikorski (World War II), **B**) A tooth of a mysterious woman from the Benedictine Abbey in Tyniec, Poland (XII-XIV century). Peaks reflect alleles determined in the HIrisPlex *loci*, which are identified on electropherograms with rs numbers or in two cases with position in amino acid chain and mutation type (insertion N29insA, nonsense mutation Y152OCH). Pull-up peaks resulted from increased fluorescence are observed at position rs1805009 in Panel B.

DNA analysis from skeletal remains may help to solve various controversies concerning historical figures. As an illustrative example, we analysed tooth S11 (Figure 1) collected from the corpse of General Władysław Sikorski. During World War II, Sikorski was Commander-in-Chief of the Polish Armed Forces and also was Prime Minister of the Polish government in exile. Sikorski died in an airplane crash at Gibraltar in 1943. His body was buried in a cemetery in Newark (UK) and after exhumation in 1993 was finally placed in the crypt of the cathedral of the Royal Castle on Wawel Hill in Kraków (Poland). Since the hypothesis that an accident had caused the airplane crush at Gibraltar was questioned, in 2008 Władysław Sikorski was again exhumed and the body was thoroughly examined [41]. Previous mitochondrial DNA analysis of the tooth was crucial for identification of General Sikorski’s remains, but a complete Identifiler STR profile and a complete Yfiler Y-STR profile were obtained too, indicating reasonably good DNA preservation [42]. A complete HIrisPlex SNP profile was generated and is shown in Figure 2. The HIrisPlex model predicted blue eye colour with high probability (*P* = 0.950) and high accuracy (99%) as well as blond hair colour (*P* = 0.852) and a light hair colour shade (*P* = 0.964) so that light blond can be assumed as the most likely hair colour (accuracy 69.5%). It is worth noting that in this particular case the eye/hair colour predictions could be confirmed with known data about Władysław Sikorski’s appearance. Although colour photographs of the General were unavailable and all known colour portraits were painted many years after his death and thus may not be considered most reliable, the fact that Władysław Sikorski indeed had blue eyes and blond hair, as predicted via HIrisPlex, was obtained from historical scripts [43].

DNA analysis of recent human skeletal remains can also be helpful in the identification of war victims. As examples, teeth S12 to S23 were collected from persons who were killed during World War II in a prison located in Poznań (Poland). In 1942, 14 skulls were sent to the Natural History Museum in Vienna and at that time they were exhibited as examples of “sub-human” skulls. In 1998, these skulls were returned to Poland, and the Institute of National Remembrance ordered examination of these human remains. In all 12 cases, besides mitochondrial DNA data, complete Minifiler STR profiles and partial Yfiler Y-STR profiles were obtained (data not shown) indicating some level of genomic DNA fragmentation. All 12 skulls were included in this study for teeth sampling. In all 12 cases, complete HIrisPlex SNP profiles were generated (Table 1) and the eye and hair colour prediction results are presented in Table 2. Among these 12 individuals, blue (probabilities 0.87 to 0.95) and brown eye colours (probabilities 0.632 to 0.711) were inferred with high accuracies (91.9% to 99%) as well as light blond, light blond/dark blond and dark blond/brown hair colours (accuracies 69.5 to 78.5%), as may be expected from individuals of (most likely) Polish European ancestry.

### Medieval samples

Analysis of DNA extracted from old bone samples is often a very challenging task due to very low amounts of DNA achieved, which additionally is very often subject to heavy degradation. Sample S24 represents a controversial case from the Benedictine Abbey in Tyniec near Kraków. During the work undertaken in the crypt of the St. Peter and Paul church belonging to the Abbey, 17 skeletons of alleged abbots were found. The burial was dated to the period of the 12^th^ to 14^th^ centuries. Unexpectedly, the anthropological examination revealed that two skeletons may be of female origin, which indeed was confirmed by DNA analysis (data not shown), while only male monks were expected. One of the two DNA samples was sufficiently preserved to enable analysis of other nuclear markers (data not shown) and was used here for HIrisPlex analysis. The mysterious woman was predicted to have dark blond/brown hair (accuracy of 78.5%) and brown eyes (accuracy of 90.4%), (Table 2 and Figure 2B). The somewhat stronger peak imbalance relative to the sample shown in the electropherogram panel A can be explained by the lower DNA quality of this medieval sample and resulting preferential amplification. Consequent higher signals observed at rs1805009 (above 8,000 RFU) led to elevated pull-up peaks. These artifacts, however, did not interfere with the phenotype interpretation from the obtained genotype data.

Samples S25 and S26 came from two skeletons revealed during conservation work conducted in the Church of St. Andrew in Kraków in 2011. The church of St. Andrew was built between 1079 and 1098, and represents a great example of the Romanesque style. Two medieval skeletons were found under the floor between the chancel and the nave of the church. Based on historical markers the grave was dated to originate from the 14th century. Further anthropological examinations indicated that the S25 male died at the approximate age of 60, whereas the S26 male was approximately 75 years old at the time of death. It is alleged that the skeletons belong to members of the Tęczyński family, representing noble Polish magnates of medieval times. The tooth collected from the deeper burial (S25) was found to be seriously affected by decay, which was reflected by a very low DNA concentration (3 pg/μl) and incomplete autosomal and Y chromosome STR profiles (NGM and Yfiler). Complete mtDNA HVI and HVII profiles were generated in both teeth (data not shown). From these data it was possible to conclude that both skeletons are of male origin and are unrelated in both maternal and paternal lines. From the partial HIrisPlex profile ascertained from S25 we successfully inferred blue eye colour (*P* = 0.899, accuracy of 95.6%), but hair colour could not be inferred because of missing genotypes at three DNA variants (N29insA, rs1805005, rs2228479). The sample S26 revealed a prediction of blond hair colour (*P* = 0.784) together with a light hair colour shade (P = 0.918) concluding that the individual had light blond hair (accuracy of 69.5%). Eye colour prediction of S26 revealed blue eyes (P = 0.919, accuracy of 97.4%) (Table 2).

The HIrisPlex system was initially designed to enable degraded DNA analysis with considering short amplicons (less than 160 bp for all amplicons) [40]. Here we showed that HIrisPlex performs successfully in degraded DNA from skeletal remains of various ages and storage conditions. However, also under such design, the possibility of allelic drop-outs and drop-ins, which have been described as typical phenomenon associated with analysis of low template DNA samples, cannot be eliminated completely. Allele drop-outs, which are explained by stochastic effects, may lead to false homozygote genotypes whereas allele drop-ins are explained by minute contaminations. Both effects may affect final results of DNA-based human identification [44,45]. Allelic drop-outs and drop-ins can also have a practical impact on EVC prediction, for example, HIrisPlex-based eye and hair colour prediction. However, the final effect depends on the particular SNP involved, as different SNPs have different impact on the final eye and hair colour prediction. Such influence may be particularly strong for strong DNA predictors, such as the IrisPlex/HIrisPlex marker rs12913832 in the *HERC2* gene, which strongly determines blue/brown eye colour. From the homozygote CC/GG genotype blue eye colour is predicted while the heterozygote CT/GA genotype most often indicate hazel/light brown eye colour [26]. A similar situation occurs in case of several DNA variants in the *MC1R* gene, such as N29insA, rs1805007 and rs1805008 that have high penetrance among red hair colour individuals. All samples analysed here were genotyped at least twice and in most cases the results were consistent. Inconsistencies were observed mainly at two DNA variants, the insertion/deletion polymorphism N29insA (first position in the assay) and the polymorphism C/A rs4959270 (15. position in the assay). N29insA was most sensitive to suboptimal quality/quantity of template DNA. The peak height is generally lower compared to the other SNPs in the HIrisPlex assay and did not reach detection limit settled at 50 RFU five times in the course of repeated analyses. It should be also mentioned that in three cases (S4, S7 and S25) this polymorphism remained undetermined (Table 1). For rs4959270, we most probably observed drop-out in two samples (S14 and S17). Table 2 shows the prediction results under the assumption of the presence of the heterozygous state CA/GT in both cases as is presented in Table 1. This discrepancy has little influence on the prediction values but affects the conclusion for S14 in terms of hair colour (light blond/dark blond for homozygote C vs. light blond for heterozygote C/A). Although drop-out was assumed as more probable, drop-in cannot be completely ruled out. Notably, these two DNA variants were also discussed by Walsh *et al.* as more sensitive than all other HIrisPlex SNPs to template DNA quantity [40]. In one of the three genotypings performed on sample S13 drop-ins were also identified at rs2402130G (position 19 of the assay), rs12896399 G (position 22) and rs683T (position 24). We assumed that these signals reflect minor contamination because they were detected only once and at significantly lower levels than other peaks in the HIrisPlex profile of this sample. Negative extraction and PCR controls were clean in case of STR analyses in this sample while in some HIrisPlex negative controls one to three peaks at a very low signal level were detected. This effect seems to be stochastic and there is no particular DNA variant in the HIrisPlex assay which can be pointed to as more prone to such minor contamination.

The results obtained here provide further evidence that quality of DNA templates from bone material depends not only on the storage time but more so on environmental conditions affecting the decomposition of the remains. Low temperature and low humidity are known to prevent DNA degradation [46], allowing successful DNA analysis even after tens of thousands of years [1]. Here we show that a complete 24 SNP HIrisPlex profile was obtained from a 12^th^ century sample (S24) collected from a skeleton that rested in relatively favourable conditions in a church. On the other hand, a partial profile was obtained from a relatively young contemporary sample (S17) that was found in open soil. However, it is difficult, as had been outlined previously [46], to draw generalizing conclusions from such data. For instance, a different 12^th^ century sample stored under expectedly similar conditions also excavated from inside a church (S25) yielded a partial HIrisPlex profile.

Obviously, special care is needed when EVC prediction is performed for samples containing DNA in suboptimal quantity and/or quality. Previous preliminary sensitivity testing of the HIrisPlex assay [40] provided an approximate threshold of 60 pg, above which allelic drop-out and drop-in were not observed. In the present study, two samples were analysed from an estimated starting template DNA amount of less than 60 pg, of which one (S24 approximately 30 pg) revealed a full HIrisPlex profile allowing both eye and hair colour prediction, while the other (S25 approximately 10 pg) lacked genotypes at three DNA variants, which, because of the particular DNA variants involved in the drop-out, did allow for eye colour, but not hair colour prediction. However, the concentration estimates established for these two samples may not be realistic as both extracts displayed signs of DNA inhibition from Quantifiler RT-PCR measurement. In any case, application of the HIrisPlex assay from low quality/quantity DNA samples, such as DNA extracted from skeletal remains or touched objects, should be accompanied with appropriate rigor, meeting recommendations settled for low template DNA samples [47].

## Conclusions

It can be anticipated that DNA prediction of EVC will soon become more widely used in genetic studies of human remains in evolutionary, anthropological and forensic investigations. The recently introduced HIrisPlex system [40] provides a convenient molecular tool for simultaneous prediction of eye and hair colour categories from DNA. As demonstrated here, the HIrisPlex system is sufficiently sensitive and robust to enable successful analysis of bones and teeth of various ages, including medieval (and expectedly older) samples. HIrisPlex can be successfully implemented in a forensic DNA laboratory equipped with the standard layout for DNA identity testing and specialized on DNA extraction from skeletal remains, and likewise in any dedicated ancient DNA laboratory. With the current study, we aim to encourage the anthropological and forensic genetic community to use the HIrisPlex system for DNA-based eye and hair colour prediction in future evolutionary, anthropological studies and for forensic case work involving skeletal remains.

## Abbreviations

aDNA: Ancient DNA; DTT: Dithiothreitol; DVI: Disaster victim identification; EDTA: Ethylenediaminetetraacetic acid; EVC: Externally visible characteristic; FDP: Forensic DNA phenotyping; HGDP-CEPH: Human genome diversity cell line panel Foundation Jean Dausset-CEPH Paris; indel: Insertion-deletion polymorphism; mtDNA: Mitochondrial DNA; NGS: Next generation DNA sequencing; RT-PCR: Real time polymerase chain reaction; SBE: Single-base extension; SDS: Sodium dodecyl sulfate; SNP: Single nucleotide polymorphism; STR: Short tandem repeat (polymorphism); WGS: Whole genome sequencing; Y-STR: Y chromosome STR.

## Competing interests

The authors declare that they have no competing interests.

## Authors’ contributions

WB and MK conceived and designed the study, provided resources, interpreted results and wrote the manuscript. JDB carried out the genotyping and elaborated results. EP participated in genotyping data analysis. SW carried out the prediction of phenotypes from genotypes and helped in manuscript writing. TK and HG provided the samples and helped to draft the case description parts of the manuscript. All authors read and approved the final manuscript.
